# Vorgaben, Zielvorstellungen, Motive, Haltungen und Denken zum ambulanten Operationsprofil der Allgemein- und Viszeralchirurgie

**DOI:** 10.1007/s00104-023-01920-y

**Published:** 2023-07-18

**Authors:** C. Paasch, C. Schildberg, M. Lehmann, F. Meyer, U. Barth

**Affiliations:** 1 Klinik für Allgemein- und Viszeralchirurgie, Universitätsklinikum Brandenburg/Havel, Medizinische Hochschule Brandenburg, Brandenburg/Havel, Deutschland; 2 Klinik für Allgemein‑, Gefäß- und Viszeralchirurgie, HELIOS Klinik Jerichower Land, Burg, Deutschland; 3 Kllinik für Allgemein‑, Viszeral‑, Gefäß- und Transplantationschirurgie, Universitätsklinikum Magdeburg A. ö. R., Leipziger Str. 44, 39118 Magdeburg, Deutschland

**Keywords:** Ambulante Operationen, AOP-Katalog, Hybrid-DRG, Ambulante Hernienchirurgie, Ambulante proktologische Chirurgie, Selektive sektorengleiche Vergütung und tagesstationäre Versorgungsformen, Surgical interventions in an outpatient clinic setting, AOP catalog, Hybrid DRG, Hernia surgery in an outpatient clinic setting, Proctological interventions in an outpatient clinic setting, Selective sector-equal reimbursement and surgical treatment in a daily-care setting

## Abstract

**Ziel:**

Das Ziel des Manuskriptes ist es, die Auswirkungen und Möglichkeiten sowie Gefahren der „Ambulantisierung“ operativer und stationärer Leistungen für die Allgemein- und Viszeralchirurgie zu erörtern und zu beurteilen.

**Methode:**

Narrative Übersicht mit Literaturbezug nach Pubmed®-Recherche unter Zuhilfenahme der Suchwörter: ambulante Operationen und stationsersetzende Eingriffe, AOP-Katalog, Hybrid-DRG, ambulante Hernienchirurgie, ambulante proktologische Chirurgie, selektive sektorengleiche Vergütung und tagesstationäre Versorgungsformen.

**Ergebnisse (Eckpunkte):**

– Im angloamerikanischen Raum wird die Versorgung von Leistenhernien hauptsächlich ambulant durchgeführt. In den USA, Schweden und Dänemark beispielsweise werden über 70 % aller Leistenhernien ambulant versorgt, in Deutschland sind es hingegen lediglich 20 %. In Deutschland definiert der Katalog ambulant durchführbarer Operationen (AOP-Katalog) und sonstiger stationsersetzender Eingriffe gemäß § 115b SGB V im Krankenhaus ambulante Eingriffe.

– Die Umsetzung der Umwandlung einer stationären in eine ambulante Leistenhernienchirurgie scheiterte bisher zudem an einem enormen Erlösunterschied. Die geplanten tagesstationären Versorgungsformen sollen nach dem Willen des Bundesgesundheitsministeriums das Pflegepersonal in den Krankenhäusern entlasten und damit die angespannte Fachkräftesituation in der Pflege reduzieren. Bis Ende März 2023 soll(te) eine spezielle sektorengleiche Vergütung, sog. Hybrid-DRGs, vereinbart werden, die unabhängig davon gilt, ob eine vergütete Leistung ambulant oder stationär erbracht wird.

– Ob eine Hernie unter stationären oder ambulanten Bedingungen durchgeführt werden kann, entscheidet sich bisher gemäß § 115b SGB V auch nach der Lokalisation dieser. Im neuen AOP-Katalog wird die Gebrechlichkeit in den Kontextfaktoren über Pflegegrad und Barthel-Index operationalisiert. Vergleicht man die Anzahl der Verschlüsselung der Prozedur 5‑530 (Verschluss Hernia inguinalis) im Jahr 2005 (184.679) mit dem Vor-Corona-Jahr 2019 (179.851), so stellt man fest, dass der Anteil der stationär versorgten Hernien annähernd über den Zeitraum von 14 Jahren gleichgeblieben ist.

– Elektive proktologische Eingriffe können in der überwiegenden Hauptanzahl ambulant durchgeführt werden. Eine stationäre Operation wird aus Gründen der Sicherheit (Blutung) und der Praktikabilität (Schmerzbehandlung, Verbandswechsel bei großen Abszessen) bevorzugt bei: ausgedehnter Hämorrhoidektomie bei massiven Befunden, großen Abszessen (z. B. Hufeisenabszessen), ausgedehnten perianalen Fistelsanierungen, insbesondere hohen trans- oder suprasphinktären Fisteln.

– Für eine flächendeckende Ambulantisierung in der Chirurgie sind Leitlinien nach dem Vorbild der „Britischen Leitlinie für ambulantes Operieren“ zu fordern. Die Einführung entsprechender Hybrid-DRGs scheint ein geeigneter Weg zu einer Kostendeckung ambulanter Operationen am Krankenhaus zu sein.

**Schlussfolgerung:**

Die Umstrukturierung der Krankenhauslandschaft und die flächendeckende Ausweitung ambulanter Operationen ist angesichts der steigenden Kosten im Gesundheitssystem und drohender Finanzierungsnöte ein unausweichliches Erfordernis, das die chirurgischen Fächer in den nächsten Jahren vor Herausforderungen stellt. Die ambulante Chirurgie wird bereits in weiten Bereichen gelebt, konnte sich aber aufgrund des Vergütungsunterschiedes bisher nicht wirklich und verlässlich durchsetzen. Hier können die sektorengleichen Pauschalen ein Ansatzpunkt sein. Des Weiteren müssen evidenzbasierte Rahmenbedingungen nach Vorbild der britischen Leitlinie für ambulantes Operieren geschaffen werden.

## Abstract (en)

**Aim:**

The aim of the manuscript is to discuss and assess the implications and opportunities as well as dangers of “outpatientization” of surgical and inpatient services for general and abdominal surgery.

**Method:**

Narrative overview with literature reference based on a PubMed search with the search terms: outpatient operations and inpatient interventions, AOP catalog, hybrid DRG, outpatient hernia surgery, outpatient proctological surgery, selective sector-equal reimbursement and day-care forms of care.

**Results (key points):**

– In the Anglo-American area, the treatment of inguinal hernias is predominantly carried out on an outpatient clinic basis. In the USA, Sweden and Denmark, for example, over 70% of all hernias are treated in an outpatient clinic setting, in Germany it is only 20%. In Germany, the catalog of operations that can be performed on an outpatient basis and other department-replacing interventions in hospitals defines outpatient interventions in accordance with § 115b Social Security Code (SGB) V (Germany).

– The conversion from inpatient to outpatient hernia surgery has also failed so far due to an enormous difference in revenues. According to the will of the Federal Ministry of Health, the planned forms of semistationary care are intended to relieve the nursing staff in the hospitals and thus relieve the tense situation of nursing professionals. By the end of March 2023, a special industry-specific reimbursement, so-called hybrid DRGs, is to be agreed, which applies regardless of whether a paid service is provided on an outpatient or inpatient basis.

– According to § 115b SGB V, whether a hernia can be performed under inpatient or outpatient conditions is also decided according to the location of the hernia. In the new AOP catalog, frailty is operationalized in the context factors via the degree of care and the Barthel index. If one compares the number of encryption procedures for the 5‑530 procedure (closure of an inguinal hernia) in 2005 (184,679) with the pre-corona year 2019 (179,851), it can be seen that the proportion of hernias treated in hospital remained approximately the same over a period of 14 years.

– Most elective proctological procedures can be performed on an outpatient basis. For reasons of safety (bleeding) and practicality (pain management, dressing change of large abscesses), inpatient surgery is preferred: extensive hemorrhoidectomy in the case of massive findings, large abscesses, extensive perianal fistula corrections, particularly high transsphincteric or suprasphincteric fistulas.

– Guidelines based on the British Guidelines for Ambulant Surgery should be required for comprehensive outpatient treatment in surgery. The introduction of corresponding hybrid DRGs seems to be the right way to cover the costs of outpatient surgery in hospitals.

**Conclusion:**

The restructuring of the hospital landscape and the nationwide expansion of outpatient operations is an unavoidable requirement in view of rising costs in the healthcare system and impending financing bottlenecks, which will pose challenges for the surgical disciplines in the years to come. Outpatient surgery is already practiced in many areas but has not become established due to the different remuneration. The flat rates for the same branches can be a starting point here. Furthermore, evidence-based framework conditions must be created along the lines of the British Guidelines for Ambulant Surgery.

## Hintergrund

Die Ausgaben für Gesundheitskosten sind trotz Einführung des DRG („diagnosis-related groups“)-Systems und Förderung des Wettbewerbs durch großflächige Privatisierungen der Kliniken in Deutschland weiter gestiegen. Da in Deutschland Patienten im Vergleich zu anderen europäischen Staaten häufiger stationär im Krankenhaus behandelt werden, liegt es nahe, dass der Fokus der aktuellen Bundesregierung auf der Verlagerung stationärer Leistungen in den ambulanten Sektor liegt. Bereits 1992 wurden die Krankenhäuser im Rahmen des Gesundheitsstrukturgesetzes zur Durchführung ambulanter Operationen nach § 115 Sozialgesetzbuch (SGB) V zugelassen. Regelungen zur Zulassung zu ambulanten Operationen, zum Leistungsumfang und zur Vergütung wurden durch die Deutsche Krankenhausgesellschaft (DKG), den GKV-Spitzenverband (GKV: Gesetzliche Krankenversicherung) und die Kassenärztliche Bundesvereinigung (KBV) im AOP-Vertrag (AOP: Ambulante Operationen und stationsersetzende Eingriffe) und einem dazugehörigen OPS-Katalog (OPS: Operationen- und Prozedurenschlüssel) ausgearbeitet. Die Operationsprozeduren und deren Abrechnung werden jährlich angepasst [1]. Dabei wurden bisher

1. Leistungen, die in der Regel ambulant zu erbringen sind (und)
2. Leistungen, die ambulant oder stationär durchgeführt werden können [1],

unterschieden.

Im Jahre 2022 veröffentlichte das Bundesgesundheitsministerium mehrere Stellungnahmen bezüglich der Reform der Krankenhausvergütung. In der Präambel heißt es dazu:

„… Die Regierungskommission ist der Auffassung, dass die bislang im DRG-System abgebildete, rein leistungs- und mengenorientierte Vergütung erhebliche Fehlanreize, insbesondere eine sachlich nicht gerechtfertigte Mengenausweitung erzeugt und die Potenziale für eine Ambulantisierung der ärztlichen und pflegerischen Versorgung nicht ausschöpft. Die Kommission geht aber zugleich davon aus, dass Leistungsanreize erhalten bleiben müssen, weil auch eine ausschließlich leistungsunabhängige Vergütung – etwa in Form eines zu 100 Prozent garantierten Budgets oder einer Selbstkostendeckung – Fehlanreize setzt und erhebliche Risiken für eine patienten- und bedarfsgerechte Versorgung sowie finanzielle Risiken für die Kostenträger auslösen würde. …“ [2].

Der AOP-Katalog für 2023 wurde vor diesem Hintergrund um 208 zusätzliche OPS-Kodes erweitert. Die stationäre Durchführung von Leistungen, die nach dem AOP-Vertrag regelhaft ambulant durchgeführt werden müssen, sollen nun anhand von Kontextfaktoren begründet werden [3].

Kontextfaktoren beinhalten bestimmte ICD-Kodes (ICD: International Classification of Diseases and Related Health Problems), bestimmte OPS-Kodes, Funktionseinschränkungen des Patienten, die Pflegebedürftigkeit nach Pflegegrad 4 oder 5, eine Beatmungszeit von > 0, Patienten bis zum ersten vollendeten Lebensjahr und Übergangsregeln für bestimmte OPS-Kodes [4]. Der aktuelle Katalog wurde erweitert, sodass nun beispielsweise die laparoskopische Leistenhernien- und die offene Nabelhernienversorgung mit Netzeinlage als auch Hämorrhoidektomien bei weniger als 3 Lokalisationen ambulant durchzuführen sind.

Das Ziel des Manuskriptes ist es, die Auswirkungen und Möglichkeiten sowie Gefahren der „Ambulantisierung“ operativer und stationärer Leistungen für die Allgemein- und Viszeralchirurgie anhand

1. einer selektiven Literaturauswahl vor allem der letzten Jahre,
2. eigener klinischer Erfahrungen (und)
3. den Empfehlungen der staatlichen Kommissionen

zu erörtern und zu beurteilen.

## Methode

Der Beitrag gibt eine narrative Übersicht mit Literaturbezug nach Pubmed®-Recherche unter Zuhilfenahme der Suchwörter: ambulante Operationen und stationsersetzende Eingriffe, AOP-Katalog, Hybrid-DRG, ambulante Hernienchirurgie, ambulante proktologische Chirurgie, selektive sektorengleiche Vergütung und tagesstationäre Versorgungsformen.

## Ergebnisse (Eckpunkte)

### Historie/Tradition

Eine Vielzahl operativer Eingriffe wurde noch bis vor 20 Jahren im Rahmen eines mehrtägigen, mehrwöchigen stationären Aufenthaltes durchgeführt. So verblieben beispielsweise Patienten nach elektiver (offener) Cholezystektomie bis zu 2 Wochen im Krankenhaus. Heute können diese in der Regel am 2. oder 3. postoperativen Tag entlassen werden. Dieser Umstand mitbegründete wahrscheinlich einen erhöhten Bedarf an Bettenkapazitäten im 20. Jahrhundert. Passend dazu wurden zwischen 1898 und 1930 beispielsweise die Heilanstalten in Berlin-Buch begründet. Dieser Krankenhauskomplex im Norden Berlins umfasste mehrere Tausend Betten für Patienten mit chirurgischen und nichtchirurgischen Krankheitsbildern.

Die Implementierung der laparoskopischen Chirurgie in den Klinikalltag der 1990er-Jahre führte bei einigen Krankheitsbildern zu einer verkürzen Liegedauer. So zeigten beispielsweise Coccolini et al. [5] in einer Metaanalyse, dass die Liegedauer um ca. 5 Tage kürzer war, wenn Patienten mit akuter Cholezystitis laparoskopisch und nicht offen-chirurgisch behandelt wurden. Dieser und andere medizinische Fortschritte wurden auch in der Hernienchirurgie erzielt. So empfiehlt die europäische Leitlinie unabhängig vom Operationsverfahren bei Patienten mit einem ASA(American Society of Anaesthesiologists)-I- und -II-Status (in einigen Fällen auch ASA III) eine ambulante Leistenhernienchirurgie. Vor diesem Hintergrund wird im angloamerikanischen Raum die Versorgung von Leistenhernien hauptsächlich ambulant durchgeführt. In den USA, Schweden und Dänemark beispielsweise werden über 70 % aller Leistenhernien ambulant versorgt, in Deutschland sind es hingegen lediglich 20 %, wobei spezielle Randbedingungen traditionell, vor Ort oder landesspezifisch zusätzlich in Betracht zu ziehen sind (z. B. naheliegende krankenhausassoziierte Hotelunterkünfte).

Im Jahre 2003 wurde in Deutschland auf gesetzlicher Grundlage des § 85 SGB V und § 17b des Krankenhausfinanzierungsgesetzes ein an Diagnosen geknüpftes Fallpauschalensystem eingeführt – die sog. German Diagnosis-Related Groups (DRG). Auf Grundlage dessen beträgt die Vergütung einer ambulanten Leistenhernienoperation trotz eingeschränkter Vergleichbarkeit der beiden Sektoren nur etwa 30–60 % der stationären Vergütung. So kann postuliert werden, dass diese Einführung möglicherweise die Bereitschaft erhöhte, zuvor ambulant durchgeführte Eingriffe nun stationär (in ca. 80 % der Fälle) durchzuführen.

### Gesetzliche Regelung und Neuerungen

#### Ambulante Operationen und stationsersetzende Eingriffe

In Deutschland definiert der *Katalog ambulant durchführbarer Operationen und sonstiger stationsersetzender Eingriffe gemäß § 115b SGB V* im Krankenhaus ambulante Eingriffe.

Mit dem MDK-Reformgesetz (MDK: „Medizinischer Dienst der Krankenkassen“) hat der Gesetzgeber eine Erweiterung des AOP-Kataloges festgelegt, um mehr stationäre Behandlungen in eine ambulante Therapie umzuwandeln. Im Auftrag der drei Selbstverwaltungspartner hat das Institut für Gesundheits- und Sozialforschung (IGES-Institut) zusammen mit dem österreichischen Gesundheitsforschungsinstitut Gesundheit Österreich ein Gutachten für einen neuen AOP-Katalog erstellt. In diesem Gutachten wurden 2476 zusätzliche medizinische Leistungen, die in den Katalog für ambulantes Operieren (AOP-Katalog) aufgenommen werden sollten, eingefügt, sodass sich die Anzahl der derzeit möglichen ambulanten Leistungen (2879 Leistungen) nahezu verdoppeln würde [6]. Der AOP-Katalog ab 01.01.2023 wurde, wie bereits erwähnt, um 208 zusätzliche OPS-Kodes erweitert. Bisher galten sog. GAEP-Kriterien („German appropriate evaluation protocol“), die eine stationäre Leistung begründeten, auch wenn diese gemäß § 115b SGB V als ambulante Behandlung vorgesehen war. Mit dem neuen AOP-Katalog ist die stationäre Durchführung von Leistungen, die nach dem AOP-Vertrag regelhaft ambulant durchgeführt werden müssen, nun anhand von Kontextfaktoren zu begründen. Diese Kontextfaktoren beinhalten bestimmte ICD-Kodes, bestimmte OPS-Kodes, Funktionseinschränkungen des Patienten, die Pflegebedürftigkeit nach Pflegegrad 4 oder 5, eine Beatmungszeit von > 0, Patienten bis zum ersten vollendeten Lebensjahr und Übergangsregeln für bestimmte OPS-Kodes [4].

Die Umsetzung der Umwandlung einer stationären in eine ambulante Leistenhernienchirurgie scheiterte bisher an einem enormen Erlösunterschied. Daher wurden der GKV-Spitzenverband, die DKG sowie die KBV verpflichtet, bis zum 31.03.2023 eine spezielle sektorengleiche Vergütung zu vereinbaren (Hybrid-DRGs), die unabhängig davon erfolgt, ob die vergütete Leistung ambulant oder stationär erbracht wird. Es soll weiterhin festgelegt werden, für welche der genannten Leistungen im nach § 115b vereinbarten AOP-Katalog die neue Vergütung Anwendung finden kann [7]. Die vereinbarte Vergütung ist individuell als Fallpauschale zu kalkulieren. Unterschiede nach dem Schweregrad der Fälle sind dabei durch die Bildung von Stufen zu berücksichtigen [7].

#### Krankenhauspflegeentlastungsgesetz – tagesstationäre Versorgungsformen

Die geplanten tagesstationären Versorgungsformen sollen nach dem Willen des Bundesgesundheitsministeriums das Pflegepersonal in den Krankenhäusern entlasten und damit die angespannte Fachkräftesituation in der Pflege reduzieren. Hier ist eine Umwandlung von vollstationären Fällen in Tagesbehandlungsfälle mit Abzug von 0,04 BWR (Bewertungsrelation) pro Nacht (ca. 140 €) bis max. 30 % der DRG geplant. Die Notwendigkeit der Umwandlung vollstationärer Behandlung in Tagesbehandlung darf durch den MDK nicht überprüft werden. Die Tagesbehandlung ist prinzipiell bei allen DRGs möglich, eine Unterbrechung für 2 Tage ist gestattet, sie beträgt mindestens 6 h und schließt AOPs, Tagesklinik, Tagesfälle ohne Einweisung, Ermächtigungen, Institutsambulanzen, Hochschulambulanzen, vor- und nachstationäre Behandlung aus. Eventuelle Fahrtkosten werden durch die Krankenkasse nicht erstattet [8].

### Spezielle sektorengleiche Vergütung

Der GKV-Spitzenverband, die DKG sowie die KBV sollen bis Ende März 2023 eine spezielle sektorengleiche Vergütung, sog. Hybrid-DRGs, vereinbaren, die unabhängig davon gilt, ob eine vergütete Leistung ambulant oder stationär erbracht wird. Zudem soll festgelegt werden, für welche der im vereinbarten AOP-Katalog genannten Leistungen die Vergütung Anwendung findet. Die vereinbarte Vergütung ist individuell als Fallpauschale gedacht, wobei Unterschiede nach dem Schweregrad der Fälle in einem Stufenkonzept berücksichtigt werden [9].

Bereits 2013 wurde auf dem Bundeskongress Chirurgie in Nürnberg ein Pilotprojekt (Thüringer Pilotprojekt) hinsichtlich der Hybrid-DRGs initiiert, welches von der Techniker Krankenkasse unterstützt wurde. Für eine ambulante Leistenbruchoperation konnten damalig ca. 600 € abgerechnet werden. Im stationären Bereich wurden dafür 2000 bis 2500 € gezahlt. Im Thüringer Pilotprojekt zahlte die Techniker Krankenkasse nun eine Hybrid-DRG in Höhe von 1600 € unabhängig davon, ob der Eingriff in einem Krankenhaus oder in einer Arztpraxis erfolgte. Seit 2017 sind 8 Kliniken und deren ambulante Einrichtungen/Praxen im Einzugsgebiet beteiligt. Nach Ausführungen des Initiators, Dr. Stephan Dittrich, im Juni 2022 konnten für das Thüringer Pilotprojekt transparente, einheitliche Qualitäts- und Honorierungskriterien für Praxen und Kliniken für identische Leistungen/Indikationen ambulant und stationär als pragmatisches Übergangsmodell geschaffen werden. Das Projekt führte demnach zu einer Beseitigung sektoraler Fehlanreize und erreichte eine stärkere Orientierung auf den Patienten [10].

Auch vor diesem Hintergrund wurde die o. g. Verhandlung im April 2023 als gescheitert erklärt. Die Hybrid-DRGs sollen nun im Rahmen der Finanzierungsreform umgesetzt werden [11].

### Elektive ambulante und stationäre Hernienversorgung in Deutschland

Ob eine Hernie unter stationären oder ambulanten Bedingungen behandelt werden kann, entscheidet sich bisher gemäß § 115b SGB V auch nach der Lokalisation dieser. So werden Zwerchfellhernien immer stationär behandelt. Gleiches galt für die minimal-invasive Kunststoffnetzimplantation bei primären und sekundären Bauchwandhernien. Bisher war es so, dass es Ausnahmen gab, die bei der Operationsplanung zu berücksichtigen waren. Zwingend ambulant müsste die Versorgung dieser Hernie im Falle des Primäreingriffes bei gesunden Patienten ohne Komorbidität erfolgen, falls die häusliche Versorgung gesichert ist. Komorbiditäten, die eine stationäre Behandlung erfordern, waren nach dem G‑AEP-Katalog neurologische Erkrankungen wie das apallische Syndrom. Klassisch waren auch Erkrankungen, die den ASA-Score des Patienten erhöhten. Dazu gehörten z. B die Herzinsuffizienz ab NYHA (New York Heart Association) 3, COPD („chronic obstructive pulmonary disease“), Gerinnungsstörungen oder auch psychiatrische Erkrankungen wie Psychosen (Tab. 1). Im neuen AOP-Katalog wird die Gebrechlichkeit in den Kontextfaktoren über Pflegegrad und Barthel-Index operationalisiert (Tab. 2; [12]).

**Table 1:** 

Erkrankung			
Neurologisch	Psychose	Apallisches Syndrom	Apoplex
Kardial	Herzinsuffizienz NYHA III	Herzklappenersatz Arrhythmie	Ausgeprägte Hypertonie
Pulmonal	Ausgeprägte COPD	Asthma bronchiale	Z. n. Transplantation
Abdominell	Leberzirrhose	Z. n. Voroperationen	Niereninsuffizienz

**Table 2:** 

ICD-Kode	Bezeichnung (2023)
U50.40	Schwere motorische Funktionseinschränkung: Barthel-Index: 20–35 Punkte
U50.41	Schwere motorische Funktionseinschränkung: Motorischer FIM: 31–42 Punkte
U50.50	Sehr schwere motorische Funktionseinschränkung: Barthel-Index: 0–15 Punkte
U50.51	Sehr schwere motorische Funktionseinschränkung: Motorischer FIM: 13–30 Punkte
U51.20	Schwere kognitive Funktionseinschränkung: Erweiterter Barthel-Index: 0–15 Punkte
U51.21	Schwere kognitive Funktionseinschränkung: Kognitiver FIM: 5–10 Punkte
U51.22	Schwere kognitive Funktionseinschränkung: MMSE: 0–16 Punkte

Um dieses festzustellen, ist es unabdingbar, dass die präoperative Evaluation des/der PatientIn auf Facharztniveau stattfindet. Natürlich ist hier nicht nur die Tatsache von Bedeutung, dass die Operationsindikation aus rechtlicher Sicht fachärztlich gestellt werden muss. Vielmehr gilt es, kompetent mit möglichst viel Erfahrung den sich vorstellenden Patienten anhand der Kontextfaktoren zu evaluieren und einzustufen, um so ggf. eine stationäre Aufnahme zu planen und vor einer (MDK-seitigen) Überprüfung abzusichern. Unabhängig von der eben genannten Berücksichtigung monetärer und wirtschaftlicher Aspekte gilt es darum, zudem das Risiko für eine Komplikation des Patienten möglichst gering zu halten, falls die Operation ambulant ohne medizinische Folgeüberwachung zu risikoreich wäre. So müssen die Bedingungen des sozialen Umfeldes des Patienten und der häuslichen Versorgung mit beachtet werden. Die Feststellung einer fehlenden häuslichen Versorgung, d. h. mindestens 24-stündigen Überwachungssituation, ist nach Auffassung einiger MDK durch Organisation einer häuslichen Versorgung durch in der Nähe wohnende Angehörige auszugleichen [13], was seitens der Leistungserbringer umstritten ist.

Teilweise wurde auch bewusst aufgrund sozialer Indikationen von den Kliniken auf Erlöse verzichtet, um eine adäquate medizinische Versorgung zu gewährleisten als auch keinerlei Abbrüche von PatientInnenbindung und -vertrauen in der (noch) wenig auf ambulante Eingriffe „trainierten“ Patientenklientel zu provozieren oder wirklich zuzulassen.

Liegen dennoch abweichend von den Kontextfaktoren medizinische oder soziale Gründe vor, die dazu führen, dass die Versorgung des Patienten in der Häuslichkeit nicht sichergestellt werden kann und dadurch der medizinische Behandlungserfolg gefährdet ist, so sind diese Gründe bei einer stationären Durchführung der Leistung fallindividuell darzustellen [4], was sicherlich heißt, dass bei Überprüfung durch den MDK die stationäre Behandlung durch das Krankenhaus zu erstreiten ist.

Zudem gibt es von Zeit zu Zeit Sondervereinbarungen mit den Krankenkassen, die eine stationäre Behandlung möglich machen. Dieses war vor allem der Tatsache geschuldet, dass Komplikationen z. B. bei intraabdominellen Eingriffen (transabdominelle präperitoneale Patch-Technik, TAPP) gravierende Folgen haben können. Natürlich war auch hier die fachärztliche Dokumentation zwingend erforderlich. Rezidivoperationen, außer offenen Operationsmethoden bei Leistenbrüchen, dürfen generell stationär behandelt werden, da hier von einem erhöhten Risiko auszugehen ist. Gleiches gilt für Notfalloperationen.

Bezüglich der Nabelhernien ist es so, dass kleine Hernien unter 1 cm, bei denen eine Primärnaht erfolgen kann, ambulant versorgt werden müssen wie auch das offene IPOM (intraperitoneale Onlay-Mesh) bei der Nabelhernie; netzbasierte Versorgungsformen sind sonst Teil des stationär zu versorgenden Kataloges. Ähnlich verhält es sich bei den anderen primären Bauchwandhernien. Für beide Gruppen gelten natürlich bei der ambulanten Versorgung oben genannte Kriterien, dass keine ausgeprägten Komorbiditäten vorhanden sein dürfen und dass die häusliche Versorgung gewährleistet sein muss. Narbenhernien müssen (so gut wie) immer netzbasiert versorgt werden und müssen damit stationär behandelt werden.

Im Falle der stationären Aufnahme der PatientInnen mit Leisten- und Femoralhernie(n) gilt dann zu beachten, dass die Patientinnen zeitnah bereits am 1. postoperativen Tag entlassen werden müssen, um die untere Grenzverweildauer (UGV) nicht zu unterschreiten. Diese beinhaltet das Risiko von Erlösabschlägen.

Auch wenn eine Operation nicht bei Vorliegen von Kontextfaktoren zwingend ambulant erfolgen muss, ist eine sorgfältige Dokumentation notwendig, um bei Rückfragen des MDK gute Gründe dafür benennen zu können. Häufig sind gerade diese Operationen, die nicht zwingend ambulant durchgeführt werden müssen, bei stationärer Aufnahme Gegenstand langwieriger Verhandlungen über die zu zahlenden Vergütungen. Leider existieren keine Studien, in denen harte Kriterien für die stationäre Aufnahme herausgearbeitet wurden. Daher gilt auch hier, wie Frings et al. es für die Varizenchirurgie formulierten, dass ein evidenzbasierter Kriterienkatalog zu fordern ist, um Transparenz, juristische Sicherheit und eine verlässliche stationäre Fallvergütung zu schaffen [14].

Aktuell wurde der Katalog dahin modifiziert, dass Leisten- und Fermoralhernien sowohl in offener als auch laparoskopischer Technik primär ambulant durchzuführen sind. Zusätzlich sind offen durchgeführte Rezidivoperationen mit Direktverschluss oder netzbasiert Teil des ambulanten Operationskataloges. Gleiches gilt für Nabelhernien, die in der offenen Technik ohne oder mit Netzeinlage (IPOM, „onlay“) ambulant operiert werden müssen, für die laparoskopische IPOM-Einlage gilt dieses nicht.

Jährlich werden in Deutschland ca. 300.000 Leistenbrüche versorgt. Eine Statistik über ambulante Hernienoperationen ist nicht zugänglich [15]. Die Operationen und Prozeduren der vollstationären PatientInnen in Krankenhäusern sind beim Statistischen Bundesamt abrufbar. Vergleicht man die Anzahl der Verschlüsselung der Prozedur 5‑530 (Verschluss Hernia inguinalis) im Jahr 2005 (184.679) mit dem Vor-Corona-Jahr 2019 (179.851), so stellt man fest, dass der Anteil der stationär versorgten Hernien annähernd über den Zeitraum von 14 Jahren gleichgeblieben ist [16].

Die internationalen Leitlinien zur Therapie von Leistenhernien bei Erwachsenen beschreiben, dass die ambulante Therapie für viele Hernien-„Repairs“ sicher, praktikabel und kosteneffizient sind. Jedoch sei unklar, welche komplexen Hernien nicht tageschirurgisch versorgt werden sollten, sodass in der Leitlinie einige Punkte zu komplexen Fällen definiert wurden:

1. Leistenhernien mit Anzeichen von Einklemmung, Strangulation, Infektion, mit relevanten präoperativen chronischen Schmerzen, schwierigen Lokalbefunden wie irreponible Skrotalhernien, (mehrfaches) Rezidiv, mit einer relevanten Vorgeschichte von Unterleibsoperationen, mit Bestrahlung und ähnlichen Befunden,
2. Leistenhernien bei Patienten mit relevanten Komorbiditäten,
3. schwierige intraoperative Befunde (und)
4. Symptome und Anzeichen für postoperative lokale Komplikationen [17].

In der zum 01.01.2024 zu erwartenden deutschen S2k-Leilinie *Hernien* sollten daher zum Punkt der ambulanten Hernienversorgung evidenzbasierte Empfehlungen nicht fehlen.

### Proktologische Operationen

Ebenso gibt es auch bei anderen Operationen eine Erweiterung. So sind elektive proktologische Eingriffe in der überwiegenden Hauptanzahl ambulant durchzuführen. Im neuen AOP-Katalog für 2023 wurden die Prozeduren

- 5‑490.1 (Inzision und Exzision von Gewebe der Perianalregion: Exzision),
- 5‑493.01 (operative Behandlung von Hämorrhoiden: Ligatur: 2 Hämorrhoiden),
- 5‑493.02 (operative Behandlung von Hämorrhoiden: Ligatur: 3 oder mehr Hämorrhoiden)

hinzugefügt. Als allgemeine Empfehlungen für ambulante proktologische Eingriffe gelten laut Kalbe et al.:

1. Grundsätzlich ambulant: Perianalvenenthrombosen, Fissurektomien, unkomplizierte Fisteln (subkutan, submukös, intersphinktär, tief transsphinktär, in Abwesenheit chronisch entzündlicher Darmerkrankungen), Abszessspaltungen, Sanierungen des Pilonidalsinus.
2. Mögliche ambulante Operation in Abhängigkeit von der individuellen Einschätzung des Blutungsrisikos (intra- und postoperativ): konventionelle Hämorrhoidektomie (Milligan-Morgan, Parks, Ferguson u. a.), staplergestützte Hämorrhoidektomien (Longo).
3. Eine stationäre Operation wird aus Gründen der Sicherheit (Blutung) und der Praktikabilität (Schmerzbehandlung, Verbandswechsel bei großen Abszessen) bevorzugt bei: ausgedehnter Hämorrhoidektomie bei massiven Befunden, großen Abszessen (z. B. Hufeisenabszessen), ausgedehnten perianalen Fistelsanierungen, insbesondere hohen trans- oder suprasphinktären Fisteln [18].

Die Inzidenz von Patienten, die sich wegen eines Hämorrhoidalleidens in ärztliche Behandlung begeben, wird auf ca. 4 % geschätzt und würde in Deutschland ca. 3,3 Mio. Behandlungsfälle betragen [19]. Die Verschlüsselung der Prozedur 5‑493 (Operative Behandlung von Hämorrhoiden) bei vollstationären Patienten in Krankenhäusern sank von 53.289 Prozeduren im Jahr 2005 auf 47.490 Prozeduren im Jahr 2019 (vor der Corona-Pandemie; [20]). Dieser Rückgang der Anzahl der Prozeduren im stationären Bereich um 11 % kann trotz aller Bemühungen der Verantwortlichen des Gesundheitssystems (noch) nicht als Trendwende bezeichnet werden.

### Intravenöse Portkatheterimplantationen

Am Beispiel der ambulant zu erbringenden i.v. Portimplantation lassen sich die strukturellen, finanziellen und personellen Probleme beispielhaft aufzeigen. Die ambulante Implantation von i.v. Portkathetersystemen hat sich in Deutschland weitestgehend etabliert. Genaue Zahlen zu den ambulant implantierten i.v. Portsystemen in den letzten Jahren sind auch hier nicht ganz verlässlich eruierbar. Hüttner et al. berichteten, dass im Jahr 2008 insgesamt 80.034 sog. TIVAP(„totally implantable venous access port“)-Implantationen bei stationären Patienten in deutschen Krankenhäusern durchgeführt wurden. Des Weiteren schätzte man eine Anzahl von mehr als 101.000 TIVAP-Implantationen im Jahr 2012 im ambulanten Bereich [21]. Die operationsbedingte Komplikationsrate liegt in erfahrenen Händen unter 2 % [22].

Beachtenswert ist in diesem Zusammenhang der Umstand, dass die i.v. Portimplantation zu den ersten Eingriffen gehört, die dem allgemeinchirurgischen Eleven am Operationstisch demonstriert und folgend assistiert werden und damit einen hohen Stellenwert im Profil von Lehroperationen innehat, aber eben im zwingenden Setting einer geforderten Erlösgenerierung dafür nicht ausreichend zeitlicher Puffer bestehen bleiben könnte bzw. ein Chirurgenteam von 2 KollegInnen für eine Vergütungskalkulation überhaupt nicht vorgesehen ist. Dies kann in der Tat als echtes Dilemma betrachtet werden.

Folgende intraoperative Komplikationen können auftreten:

- Blutung/Hämatom,
- arterielle Fehlpunktion,
- Pleuraverletzung,
- Pneumo‑/Hämatothorax,
- Gefäß‑, Vorhof- oder Ventrikelperforation (Führungsdraht),
- kardiale Arrhythmie,
- Perikardtamponade,
- Luftembolie (und),
- Verletzung/Irritation des Plexus brachialis [3].

Postoperative Probleme können sein:

- Nachblutung,
- Wundinfektion/-heilungsstörung,
- Hypergranulation,
- Katheterfehllage,
- i.v. portkatheterassoziierte venöse Thrombosierung,
- i.v. Portdysfunktion
  (bei i.v. Portkammerpunktion, Blutaspiration, Spülinjektion, Verumgabe, Infusion),
- i.v. Portkammerlageveränderung/‑migration,
- Hautemphysem,
- trophische Störungen/Hautperforation im i.v. Portkammerbereich (und)
- i.v. Portinfekton (Hautareal, Kammer, Katheter).

Damit ist dem perioperativen Management besondere Beachtung bei immunsupprimierten PatientInnen und jenen mit Gerinnungsstörungen als auch, in Anbetracht der wenn auch nicht hohen Komplikationshäufigkeit, aber doch des nicht gerade schmalen Komplikationsprofils, der Aufklärung außerordentlich Sorgfalt zu schenken. Auch die ambulante Implantation von i.v. Portsystemen ist je nach Kategorie des Krankenhauses ebenso mit erheblichen Kosten verbunden, die meist nicht von den im EBM (Einheitlicher Bewertungsmaßstab) erzielten Erlösen in adäquater Weise gedeckt werden. So konnten Petersen et al. bereits 2013 für ambulante i.v. Portimplantationen am Universitätsklinikum Magdeburg keine Kosteneffektivität in detaillierter Kostenaufschlüsselung (Personal, Material, Operationssaalminute) nachweisen [23]. Hinzu kommt, dass vereinzelt Krankenkassen in einer i.v. Portimplantation eine vorbereitende Maßnahme für einen späteren stationären Aufenthalt im Zuge der stationären Chemotherapie sehen und eine separate Vergütung nach dem AOP-Vertrag verweigern und den ambulanten Eingriff als vorstationäre Leistung werten, der in der stationären Fallpauschale enthalten wäre. Die vorstationäre Behandlung ist auf längstens drei Behandlungstage innerhalb von fünf Tagen vor Beginn der stationären Behandlung begrenzt. Für außerhalb dieser Frist durchgeführte ambulante Operationen dürfte es hingegen keine Einschränkung der Abrechnung als ambulante Operation nach dem AOP-Katalog geben [24].

### Tagesstationäre Versorgungsformen

Voraussetzungen für eine tagesstationäre Versorgung in der Chirurgie sind eine

- intakte und ausreichende Infrastruktur,
- gute Patientencompliance (und)
- speziell dafür eingerichtete Personal- und Versorgungsstruktur im Krankenhaus.

Da die Fahrtkosten zum und vom Krankenhaus nach Hause nicht von der Kasse übernommen werden sollen, ist eine Umsetzung in ländlichen Regionen nahezu unmöglich. Zudem dürfen die tagesstationären Behandlungen nicht mit ambulanten Operationen, Tagesklinikaufenthalten, Tagesfällen ohne Einweisung, Behandlung im Rahmen von Ermächtigungen, in Institutsambulanzen, Hochschulambulanzen sowie mit vor- und nachstationären Behandlungen kollidieren [8]. Die Anzahl der Patienten und Behandlungen, die sich in chirurgischen Fächern dafür eignen, ist als marginal einzuschätzen und wird (eher) nicht zu der gewünschten Entlastung des Pflegepersonals führen.

### Spezielle sektorengleiche Vergütung

Ein Zweiphasenkonzept für ein einheitliches und sektorengleiches Vergütungssystem wurde vom Hamburger Center for Health Economics (HCHE) in Zusammenarbeit mit der TU Berlin, dem Zentralinstitut für kassenärztliche Versorgung (ZI), dem Deutschen Krankenhausinstitut (DKI) und dem BKK-Dachverband erarbeitet. Ausgehend vom AOP-Katalog, sollen in Phase 1 sektorengleiche Leistungsgruppen (SLG) auf Basis des bestehenden Kostenrahmens des Instituts für Entgeldsystem im Krankenhaus (InEK), bereinigt um die ausschließlich stationär anfallenden Kosten, berechnet und über sog. sektorengleiche Pauschalen (SP) vergütet werden. In einer Übergangsphase von 3 Jahren soll weiter eine sektorengleiche Datengrundlage geschaffen werden. Auf dieser Datengrundlage werden Leistungskomplexe kalkuliert und empirisch bewertet. Nach einem Baukastenprinzip werden aus den Leistungskomplexen flexibel zusammensetzbare sektorengleiche Leistungsgruppen (SLG) zusammengestellt und über sektorengleiche Pauschalen (SP) vergütet [25].

Im Gutachten zur Identifizierung einer initialen Auswahl von Leistungsbereichen für eine sektorengleiche Vergütung wurden Leistungsbereiche ausgewählt, die Teil des AOP-Katalogs sind, eine geringe Verweildauer aufweisen und durch ein hohes stationäres Fallzahlvolumen charakterisiert sind [26]. Für die Allgemein- und Viszeralchirurgie bedeutsam wurden die OPS-Kodes

- 5‑530/1/4/6 (Verschluss der Hernie),
- 1‑650 (diagnostische Koloskopie),
- 4‑452 (lokale Exzision […] Dickdarm),
- 5‑513/526 (Endoskopische Operation Galle/Pankreas),
- 5‑490/2/3 (Inzision […] Perianalregion/Analkanal/Operation Hämorrhoiden),
- 1‑640/1/2 (diagnostische retrograde Darstellung Galle/Pankreas) und
- 5‑897 (Exzision […] Sinus pilonidalis)

identifiziert [26].

## Diskussion

Die Ambulantisierung in Deutschland wird weiter vorangetrieben. Dem stehen durchaus zahlreiche objektive Aspekte gegenüber, die eine ambulante Versorgung ohne Qualitätseinbuße oder Patientengefährdung nicht erlauben (Tab. 3). Der Gesetzgeber hat daher den zuständigen Selbstverwaltungsorganen Zeitmarken gesetzt. Vorbereitende Leitlinien oder evidenzbasierte Empfehlungen der einzelnen Fachgesellschaften wurden in den vergangenen Jahren kaum diskutiert, da sicherlich die Auswirkungen der Corona-Pandemie das tägliche Geschehen überlagerten. Doch jetzt sind die verantwortlichen ChirurgInnen in den Kliniken in zeitlichem Zugzwang, um sich auf die geplanten Veränderungen vorzubereiten. Zunächst müssen die Kliniken in Vorleistung gehen, um ihre ambulanten Strukturen auszubauen. Die Integration ambulanter Operationen in einem größeren Ausmaß in die Planung bisheriger Operationskapazitäten mit geeigneten postoperativen Versorgungsstrukturen für ambulante Patienten erfordert gerade in kleineren Häusern An- und Umbauten, die neben Planung und Durchführung auch einer Finanzierung bedürfen. Ganz klar ist, dass für eine flächendeckende Ambulantisierung in der Chirurgie Leitlinien nach dem Vorbild der *Britischen Leitlinie für ambulantes Operieren* zu fordern sind.

**Table 3:** 

*Patientenbezogen*	Alter, u. a.		
	Anamnese: z. B.	Narkosezwischenfall	
		Herzstillstand	
		Blutungskomplikation	
		Z. n. notwendiger ECMO-Behandlung	
		Z. n. Lungenembolie	
		Z. n. Intubationsproblem	
	Nebenerkrankungen	Hämophilie	
		Thrombophilie	
		Demenz	
		Herzinsuffizienz ab NYHA 3	
		COPD	
		Gerinnungsstörungen	
		Psychiatrische Erkrankungen wie Psychosen	
		Immunsuppression	
		Gebrechlichkeit (über Pflegegrad und Barthel-Index)	
		Organfunktionsstörungen	
		> ASA III	
	(Abdominelle) Voroperationen		
	Vorbehandlungen (z. B. Radiatio)		
	Chronische Schmerzen		
	Funktionseinschränkungen des Patienten		
	Einschränkung der Mobilität		
	Pflegebedürftigkeit nach Pflegegrad 4 und 5		
	Fehlende Compliance		
	Patienten bis zum ersten vollendeten Lebensjahr		
	Individueller Wunsch		
*Eher befundassoziiert*	Kritische Ischämie		
	Komplexe Läsionen		
	Narbenhernie (da stets netzbasierte Versorgung)		
	Nabelhernie > 1 cm		
	Irreponibilität:	Eingeklemmte Hernie	
		Inkarzerierte Hernie	
		Neigung zur Strangulation des Hernieninhalts	
	Rezidivsituation		
*Begleitfaktoren*	Medikation	Thrombozytenaggregationshemmer	
		Antikoagulation:	NOAKs
			Vit.-K-Antagonisten
		Immunsuppressiva	
	Polypharmazie		
	Ausgeprägte Komorbiditäten (s. oben)		
*Eingriffsimmanent* (verfahrensabhängig)	Hohe Dringlichkeit:	Notfallversorgung	
		Versorgung in aufgeschobener Dringlichkeit	
	Rezidivhernienoperationen		
	Netzbasierte Versorgungsformen von Hernien		
	Gefäßschleusen > 7 Fr		
	Intraoperative Probleme	Narkose	
		Beatmung > 0	
		Blutung(sneigung)	
*Umfeldrelevant*	Telefon (funktionierend)		
	Entfernung der Angehörige		
	Häusliche Versorgung nicht gewährleistet		
*Erlöspotenzial*	Fehlende lokale Wirtschaftlichkeitsprüfung		
	Übergangsregeln für bestimmte OPS-Kodes		
	*[Katalog amb. durchführbarer Leistungen]*		
*Juristische Sicherheit*	Aussichtsreich zu gewährleisten		
*Verlässliche stationäre Fallvergütung*			

Hier werden aus langjähriger Erfahrung Richtlinien und Empfehlungen vorgegeben, die an die

- Anforderungen des ambulanten Operierens im Wandel der Jahre,
- Bandbreite der durchgeführten Eingriffe (als auch)
- Zusammensetzung des Patientenkollektivs im Hinblick auf Altersstruktur und Komorbiditäten

regelmäßig angepasst werden [27]. Nur auf Grundlage eines strukturierten und evidenzbasierten Kriterienkataloges, der Transparenz, juristische Sicherheit und eine verlässliche Fallvergütung schafft [13], sind die gesundheitspolitischen Vorgaben mit einer entsprechenden Qualität und Sicherheit für den Patienten zu bewältigen. Die Einführung entsprechender Hybrid-DRGs scheint der richtige Weg zu einer Kostendeckung ambulanter Operationen am Krankenhaus zu sein. Jedoch sollte zuvor eine lokale Wirtschaftlichkeitsprüfung der ambulanten operativen Versorgung erfolgen, um zu sehen, ob die Kosten tatsächlich durch diese DRGs gedeckt werden können.

Eine weitere Problematik ergibt sich aus der von der Bundesregierung geplanten Umstrukturierung der Krankenhauslandschaft, die mit einer Abstufung in 3 Krankenhauslevel geplant ist, wobei Level I und III nochmals differenziert werden. Im Level-I-Krankenhaus ist die besondere Verzahnung von ambulanter und stationärer Krankenhausbehandlung geplant. Dem universitären Level IIIU kommt eine Sonderrolle mit erweitertem Leistungsspektrum und Zusatzaufgaben zu, insbesondere bei der Koordination der regionalen Versorgung und der Unterstützung der anderen Krankenhäuser über Zentren und Telemedizin [2]. Die Bedingungen, Strukturen und Kostendeckung einer Ambulantisierung an Universitätskliniken sind dann gesondert zu prüfen. Petersen et al. konnten bereits 2013 für ambulante Hernienoperationen und i.v. Portimplantationen am Universitätsklinikum Magdeburg keine Kosteneffektivität nachweisen [23]. Weiterführende detaillierte Kostenbetrachtungen und -berechnungen sind erforderlich, um Kostentreiber (z. B. für subspezialisierte Interventionen ausgestattete Operationssäle mit höchstem Stundensatz für ambulante Eingriffe „missbräuchlich“ zu nutzen) zu eruieren und realitätsnahe Erörterungen zu führen und praxisrelevante Regelungen zu treffen, zu optimieren und anzupassen.

Andererseits sollte darauf geachtet werden, dass Sondervereinbarungen beispielsweise für Universitätskliniken nicht kleinere Krankenhäuser benachteiligen. Eine künftige Leitlinie muss dann für alle gelten. Wenn größere Kliniken aufgrund fehlender ambulanter Strukturen oder zu Ausbildungszwecken ambulante Eingriffe stationär behandeln und dafür nicht sanktioniert werden, würde diese Benachteiligung stattfinden.

Ein dahingehend aber offenes Thema stellt die Realisierung humanmedizinischer und fachdisziplinspezifischer Lehre sowie Aus- und Weiterbildung dar im Zuge von eng getakteten organisatorischen Abläufen, effektiver Personalbesetzung als auch zeitsparenden Operations- bzw. Interventionszeiten mit Facharztstandard, ein ungelöst erscheinendes Problem [25].

Gegenüber einem überambitioniert forcierten Bestreben, Operationen zu ambulantisieren (Cholezystektomie, Schilddrüsenresektion etc.), ist eine fachlich zu begründende Zurückhaltung angezeigt.

Zudem sollte berücksichtigt werden, dass durch eine übertriebene unter dem Deckmantel der Kosteneinsparung vorangetriebene Ambulantisierung die chirurgische Entscheidungsfreiheit zumindest eingeschränkt werden kann. Dabei ist besonders in Rechnung zu stellen, dass die Kategorisierung in ambulante vs. stationäre Operationen nicht die individuelle fachärztliche Entscheidung berücksichtigt, die auf der klinischen Erfahrung und der persönlichen klinischen Untersuchung mit Anamneseerhebung beruht.

Unseres Erachtens kann dieses durch ein „abstraktes“ Einteilungssystem nur eingeschränkt abgebildet werden, da diese Vorgänge sehr komplex sind.

In der Zusammenschau scheint es unabdingbar, diesen nun initiierten Prozess der forcierten Ambulantisierung kontinuierlich evaluierend zu begleiten. Dieses Monitoring sollte auch i) repräsentative Umfragen einschließen, in denen der Arbeitsaufwand der Pflege erfasst wird, oder ii) abzuleitende Forschungsfragen für die klinische Versorgungsforschung (wie z. B. zu Qualitätskriterien). Die 1. postoperative Visite findet schließlich weiterhin statt, nur eben ambulant. Hier ist eine bloße Verlagerung des gleichbleibenden Gesamtaufwandes der Betreuung in den ambulanten Sektor zu erwarten. Zudem ist es denkbar, dass die Betten künftig mit pflegeintensiveren Patienten belegt werden und die Belastung der Pflege dadurch steigt. Dies ist wahrscheinlich ein erwünschter Effekt, der jedoch aus der Perspektive der Autoren nur tragbar ist, wenn gleichzeitig der Stellenschlüssel angehoben wird. Dann stellt sich die Frage, wie man diese Stellen besetzen kann vor dem Hintergrund des Fachkräftemangels. Es ist anzunehmen, dass kleine Krankenhäuser wirtschaftlich unter den neuen Rahmenbedingungen nicht überlebensfähig sind. Dies könnte vielleicht dazu führen, dass das dann dort entlassene Pflegepersonal in Krankenhäusern der Level-II- und -III-Versorgung arbeitet. Aber, ob dann diese Berufsgruppe, auch die ärztliche, bereit ist, zum neuen Arbeitgeber zu pendeln, ist fraglich. Es gibt im ambulanten Pflegesektor schließlich auch im ländlichen Bereich eine Vielzahl offener Stellen.

Als limitierend für diese Übersicht ist aktuell hervorzuheben, dass bisher noch nicht geklärt ist, ob diese Beschlüsse in voller Gänze praktikabel sind. Es ist somit denkbar, dass eine Adaption an die Verhältnisse in der Realität noch erfolgen muss. Dieses wird jedoch die Zukunft zeigen.

Abschließend seien die *konkreten Lösungsansätze* noch einmal zusammenfassend aufgelistet:

- Bezug zu geeigneten Leitlinieninhalten anderer Länder mit hohem Erfahrungswert und Nutzungsgrad ambulanter Operationen.
- Nur auf Grundlage eines strukturierten und evidenzbasierten Kriterienkataloges (z. B. einheitliche Qualitäts- und Honorierungskriterien für Praxen und Kliniken für identische Leistungen/Indikationen ambulant und stationär), der Transparenz, juristische Sicherheit und eine verlässliche Fallvergütung schafft [13], sind die gesundheitspolitischen Vorgaben mit einer entsprechenden Qualität und Sicherheit für den Patienten zu bewältigen.
- (Noch ausstehende und bis zum 31.03.2023 eigentlich zu vereinbarende) Hybrid-DRGs
  ➔ Nach Scheitern sollen nun die Hybrid-DRGs im Rahmen der Finanzierungsreform umgesetzt werden.
- Sektorengleiche Vergütungen, die den bisherig erlöstechnischen Nachteil einer ambulanten Versorgung abbauen und mehr effektiven Anreiz generieren helfen, im eher ambulanten Setting tätig zu werden.
  ➔ Beseitigung sektoraler Fehlanreize und damit eine wünschenswert stärkere Orientierung auf den Patienten.
  ➔ Es wird festgelegt, für welche der im vereinbarten AOP-Katalog genannten Leistungen die Vergütung Anwendung findet.
  ➔ Die vereinbarte Vergütung ist individuell als Fallpauschale gedacht, wobei Unterschiede nach dem Schweregrad der Fälle in einem Stufenkonzept berücksichtigt werden [9].
  ➔ In 3 Jahren Übergangsphase soll weiter eine sektorengleiche Datengrundlage geschaffen werden, worauf Leistungskomplexe kalkuliert und empirisch bewertet werden – nach einem Baukastenprinzip werden aus den Leistungskomplexen flexibel zusammensetzbare sektorengleiche Leistungsgruppen (SLG) zusammengestellt und über sektorengleiche Pauschalen (SP) vergütet [25].
- Einführung von Krankenhausversorgungsstufen: Im Level-I-Krankenhaus ist die besondere Verzahnung von ambulanter und stationärer Krankenhausbehandlung geplant.
- Weiterführende detaillierte Kostenbetrachtungen und -berechnungen sind erforderlich, um Kostentreiber (z. B. für subspezialisierte Interventionen ausgestattete Operationssäle mit höchstem Stundensatz für ambulante Eingriffe „missbräuchlich“ zu nutzen) zu eruieren.

## Fazit

Die Umstrukturierung der Krankenhauslandschaft und die flächendeckende Ausweitung ambulanter Operationen ist angesichts der immens steigenden Kosten im Gesundheitssystem und drohender Finanzierungsnöte nicht nur politischer Wille, sondern auch ein unausweichliches Erfordernis. Dies stellt die chirurgischen Fächer in den nächsten Jahren vor große Herausforderungen.

Die ambulante Chirurgie wird bereits in weiten Bereichen gelebt, konnte sich aber aufgrund des Vergütungsunterschiedes bisher nicht wirklich entscheidend durchsetzen bzw. erlöstechnisch wirksam umsetzen lassen. Hier können die sektorengleichen Pauschalen ein Ansatzpunkt sein. Des Weiteren müssen evidenzbasierte Rahmenbedingungen nach dem Vorbild der *Britischen Leitlinie für ambulantes Operieren* geschaffen werden, um Transparenz und juristische Sicherheit zu schaffen.

Tagesstationäre Behandlungen in der Chirurgie werden eher die Ausnahme bilden und wohl nicht zur gewünschten Entlastung der angespannten Situation im Pflegebereich beitragen.

Ein systematisches Monitoring dieses Prozesses auch in Form repräsentativer Umfragen oder abzuleitender Forschungsfragen für die klinische Versorgungsforschung (wie z. B. Qualitätskriterien) ist erforderlich, um etwaige Korrekturen durchführen zu können.

Dennoch – bei aller Einsicht und gegebenem Verständnis für anstehende Erfordernisse – ist das Ziel ärztlichen Handelns in erster Linie zu helfen und nicht zu schaden – *nicht Kosten zu sparen*. Diese Prämisse sollte für Ärzte und Wissenschaftler nicht hintenangestellt werden – sie sollten sich nicht zum Erfüllungsgehilfen einer Initiative machen lassen, die in erster Linie Kosten vermeiden soll!
